# Endovascular Treatment of Cerebral Mycotic Aneurysm: A Review of the Literature and Single Center Experience

**DOI:** 10.1155/2013/151643

**Published:** 2013-12-09

**Authors:** Mario Zanaty, Nohra Chalouhi, Robert M. Starke, Stavropoula Tjoumakaris, L. Fernando Gonzalez, David Hasan, Robert Rosenwasser, Pascal Jabbour

**Affiliations:** ^1^Department of Neurosurgery, Thomas Jefferson University, Jefferson Hospital for Neuroscience, Philadelphia, PA, USA; ^2^Department of Neurological Surgery, University of Virginia School of Medicine, Charlottesville, VA, USA; ^3^Department of Neurosurgery, University of Iowa, Iowa City, IA, USA; ^4^Division of Neurovascular Surgery and Endovascular Neurosurgery, Department of Neurological Surgery, Thomas Jefferson University Hospital, 901 Walnut Street 3rd Floor, Philadelphia, PA 19107, USA

## Abstract

The management of mycotic aneurysm has always been subject to controversy. The aim of this paper is to review the literature on the intracranial infected aneurysm from pathogenesis till management while focusing mainly on the endovascular interventions. This novel solution seems to provide additional benefits and long-term favorable outcomes.

## 1. Introduction

Intracranial infectious aneurysms (IIAs) or mycotic aneurysms are a rare entity and represent 0.7 to 5.4% of all cerebral aneurysms [1]. The name mycotic originated from the fact of their resemblance to fungal vegetation [2]. Although they can be caused by fungal pathogen, they are most commonly due to bacterial infection [3]. Historically the management of mycotic aneurysms relied on surgery and antibiotics with limited use of endovascular therapy fearing the risk of overwhelming infection by introducing a foreign body to an infected region [4]. This theoretical fear exists in spite of the absence of reports in the literature on persistent infection or abscesses formation following endovascular surgery [5]. A recent review of the literature that examined 287 cases of cerebral mycotic aneurysms (CMAs) [5] found no postprocedural infection in the 46 cases treated by endovascular coiling. In another study, coiling was successful even in the presence of active bacteremia [6]. However, the safety and efficacy of these techniques are published in case-series and case-reports. Therefore, endovascular treatment remains an individualized therapy with no standard guidelines [7]. Given the inconsistency in IIA's evolution and response to treatment and given the lack of randomized controlled trials (RCTs), there has not been any widely accepted standard management [5]. The purpose of this paper is to briefly review cerebral mycotic aneurysms while focusing on the endovascular approach for their management.

## 2. Methodology

We performed a literature review using MEDLINE. The following meshwork words were used individually or in combination: mycotic, cerebral, infectious, intracranial, aneurysm, endovascular, treatment, management, and Onyx. We managed to find 3 articles on the use of Onyx in the treatment of IIAs. Other articles were included in our study using a more extensive search to briefly review the pathogenesis of the disease and to evaluate other alternative managements. The search was limited to the studies published in English.

## 3. Epidemiology

IIAs represent 5% of all intracranial aneurysms [8]. Currently there are no rigorous population-based epidemiological studies, but an analysis of a pooled cohort by Ducruet et al. revealed that 65% of patients with IIA have an underlying endocarditis [5]. The prevalence has decreased from 86% after the advent of antibiotic era [9]. The most common sources of infectious bacteremia remain to be intravenous (IV) drug abuse and poor dental hygiene. Direct invasion of the vascular wall from a nearby infectious focus, such as cavernous sinus thrombophlebitis and bacterial meningitis, is also common cause of IAA. The median age tend to vary depending on the reviews between 35.1 [5] and 53 years [10]. Some studies reported a higher male predominance while the pooled cohort done by Ducruet et al. showed similar proportions of both genders (52% males and 48% females) [5].

## 4. Pathology and Pathogenesis

The process is the result of a developing infectious process involving the arterial wall [11]. The acute inflammation leads to neutrophils infiltration followed by degradation of the media and adventitia, fragmentation of the internal elastic lamina and proliferation of the intima. The weakened vessel wall in combination with the pulsatile pressure in the vasculature leads to an aneurysm formation and consequential growth [5]. Most of the authors prefer the term pseudoaneurysm [12], although both are widely used. Many processes may contribute to the development of IIAs: septic emboli lodging at distal branches, spreading infection involving the vasa vasorum, and periarterial lymphatic and vascular manipulation precipitating infection [2], all of which can lead to focal polymorphic neutrophil infiltration with enzymes and proinflammatory cytokine secretions. Consequently, the inflammatory reaction contributes to vessel friability, weakening, and pseudoaneurysm formation. Grossly, the aneurysm appears friable, having a thin-wall and wide or absent neck. This predisposes the aneurysm to rupture and consequent bleeding. If it ruptures, the mortality rate can be extreme, as high as 80% [13, 14]. Even though a fusiform morphology points toward a mycotic pseudoaneurysm, a saccular morphology does not exclude it, as it has been shown that approximately 41% of mycotic aneurysm in the literature are saccular [5].

Even though virus and fungi can cause IIAs, bacterial infection remains by far the most predominant cause. The most commonly reported bacterial pathogens are *S. aureus* and *Streptococcus* species. IIAs have been described following viral infection such as HIV-1 and VZV [15, 16] and fungal infection such as *Candida* and *Aspergillus* [4]. IIAs can be formed at distal branching points when the infectious agent spreads by hematogenous route, as seen in endocarditis, or it can be formed near the infected foci when the infectious agent spreads by direct invasion of the arterial wall from the extravascular site [5, 9]. The latter is more commonly seen in immunocompromised patients [9, 17]. The most common location of IIA seems to be the anterior circulation, mainly the MCA and its distal branches, contributing to as much as 50–78% of all IIAs [4, 5, 9].

## 5. Clinical Manifestations and Diagnosis

IIA's natural history is somewhat unpredictable but linked to significant mortality ranging from 30% to 80% if rupture occurs [18]. Some studies reported rupture as the most common presentation of IIAs, and most of the studies reported that headache followed by fever is the most common symptom [18]. However, a recent review found septic infarct to be more common than intraparenchymal hemorrhage (IPH) and focal neurologic deficit to be a more common initial presentation than fever [5]. The bleeding can be subarachnoid, intraparenchymal, or intraventricular [5]. Other signs and symptoms of IIAs are due to the underlying etiology [19], such as septic emboli, fever, and chills, or to the mass effect of the aneurysm. Silent IIAs are not uncommon and can represent up to 10% of autopsy cases [20]. It is noteworthy that in contrast to saccular aneurysm, size does not seem to predict the risk of rupture [21]. When the CMA is extracranial, the presentation tends to be different. When this is the case, the most common presentation is a pulsatile painful lateral cervical mass, which may compress the cranial nerves resulting in dysphagia and dysphonia [22]. If it is left untreated, it may rupture causing a hemorrhagic shock or may deliver septic emboli to the anterior circulation of the brain [22].

The diagnosis of mycotic aneurysms relies on the presence of a predisposing infectious process with an aneurysm documented by vascular imaging. Some pieces of literature even recommend screening patient with bacterial endocarditis for intracranial aneurysms given the strong correlation between the two [5]. Digital subtraction angiography (DSA) continues to be the gold standard for the diagnosis of IIA [20], although CT angiography and magnetic resonance imaging can be used [5]. Some of the findings on DSA that points toward IIA are the fusiform shape, the multiplicity, the distal location, and the change in size on follow-up angiography [5]. Positive culture from the wall itself can confirm the diagnosis [5]. Other indicators are positive blood culture (only found in 35.6%), leukocytosis, elevated erythrocyte sedimentation rate (ESR), and elevated C-reactive protein (CRP) [5].

## 6. Treatment

### 6.1. Approach to Management

Given the lack of RCTs, there are currently no standards to guide clinical decision-making. Treatment involves antimicrobial agents, surgery, endovascular approach, and/or a combination of them [9]. As a rule, IIAs management depends essentially on whether it has ruptured or not [9], the aneurysm characteristics, and the overall health status of the patient.

For unruptured IIAs in patients with high surgical risk, conservative treatment with antibiotic is the mainstay therapy. Antibiotics are guided by blood and cerebrospinal fluid (CSF) cultures. If the results were negative, empiric treatment based on suspected pathogens is continued. A period of four to six weeks of antimicrobial therapy is generally recommended [23]. An aneurysm has a high surgical risk if there is a circumferential vessel involvement, if the location is proximal, or if parent artery sacrifice cannot be done due to considerable neurological deficits. These characteristics render the surgery or the endovascular therapy difficult and unsafe. Follow-up angiography is necessary to assess the risk of rupture, which is always present even with appropriate medical therapy [5]. Conservative management yields different outcomes in terms of change in size or disappearance of the aneurysm. The outcome with conservative management is worse than that of invasive treatment when the latter is indicated [20, 24].  Table 1 summarizes some of the outcomes after conservative management. Resistance to conservative treatment is suspected when the aneurysm size increases or remains the same and/or when other aneurysms develop while the patient is on the appropriate antibiotics. In this case, invasive management is warranted [1, 9]. However, some authors advise for endovascular or surgical management whenever the aneurysm is accessible [21], regardless of the rupture status.

In the case of unruptured aneurysm without high surgical risk, endovascular or surgical treatment is advised irrespectively of the size because of the high risk of rupture and the weak association between size and rupture [21].

Ruptured aneurysms on the other hand should be immediately secured by surgical or endovascular means. The success of endovascular or surgical treatment depends mostly on the aneurysm morphology, the comorbidities of the patient, and the presence of an associated intracerebral hemorrhage [25]. The choice between endovascular and open surgery is complex and should be individualized.

### 6.2. Surgical Management

A good candidate for surgery would be a young symptomatic patient with surgically accessible IIA and/or when a significant hematoma with mass effect is present [9]. Open surgery however would be challenging when the location of the aneurysm is in the distal anterior circulation. From a technical point of view, clipping a mycotic aneurysm is more difficult than a regular saccular aneurysm due to the friable nature of the aneurysm and the absence or the deformity of the neck. In addition, localizing a distal branch aneurysm might be challenging. However, image guidance technology may help in that issue. Open surgery faces a major limitation when the patient is candidate for cardiothoracic surgery, which requires heparinization and anticoagulation. This puts the patient at higher risk of intracranial bleeding after craniotomy. Even more, studies have shown that cardiothoracic surgery following craniotomy increases the risk of perioperative heart failure [26–28]. The major complications of surgery are perioperative rupture and clip erosion of the parent artery [7, 29]. An alternative option in an unruptured aneurysm to delay surgery and give adequate time for the aneurysm to become fibrotic, minimizing therefore the risk of perioperative rupture and enabling direct clipping [5]. Even then, the risk of surgery remains high [5]. For all the previous reasons and given that many patients with IIA are quite ill and have multiple comorbidities, surgery is falling out of favor [29]. In these settings, the endovascular option seems to replace surgery as standard of care in treatment of IIAs [29], yet the optimal treatment paradigm remains controversial.

### 6.3. Endovascular Management

Endovascular techniques are rapidly gaining ground in the management of all types of cerebral aneurysms [30–41]. For mycotic lesions, the advantages of endovascular therapy over surgery are a decreased risk of anesthesia particularly in patients with impaired valve function, rapid institution of anticoagulation therapy, and shortening of the delay between aneurysm treatment and cardiac surgery. The delay can be reduced from 2-3 weeks to as little as 1 day [5, 9, 25, 27]. A major indication for endovascular therapy would be a patient with high surgical risk, a patient candidate for cardiac surgery [5], and a surgically inaccessible or multiple IIAs [42].

Current strategies in endovascular therapy include an indirect approach by parent artery occlusion (PAO) using coils or liquid embolic agents (LEAs) and direct approach by embolization of the aneurysm using coils, stent-assisted coiling (SAC), flow diverters, and LEAs [7, 43, 44]. PAO is attempted when the aneurysm is distally located, dysplastic, involving the whole circumference of the parent vessel, and having a complex morphology, provided that the area of the brain supplied by that artery is noneloquent. Intracranial balloon test occlusion or amobarbital injection testing can help determining whether the area is eloquent or not when the provider is unsure [7]. IIAs that are proximal in location such as those arising from cavernous ICA tend to be more treated by a direct approach, while both approaches are equally used for aneurysms that are distal in location such as those arising from MCA and posterior cerebral artery (PCA). When the aneurysm is difficult to reach, LEAs can be used for distal PAO (N-butyl 2-cyanoacrylate, NBCA, ethylen-vinyl alcohol copolymer, Onyx). The advantages and disadvantages of the different agents used are summarized in Table 2.

Endovascular coiling has been attempted by Andreou et al. [10] and Chapot et al. [42] with successful occlusion, without any rupture or death (Table 3) [1, 42, 45]. Sugg et al. [25] presented a case-report in which an IIA was treated by Neuroform stent. The major drawback was the use of antiplatelet agents [27], which can be critical if the aneurysm ruptured. Jadhav et al. [29] used Onyx 18 to treat 2 cases of mycotic aneurysm, one due to its resistance to antibiotic treatment and the other due to its high risk of rupture in the setting of chronic anticoagulation in a patient with antiphospholipid syndrome [29]. Onyx has the advantage over NBCA of being nonadhesive, with a long precipitation time. This allows for more precise control resulting in more satisfactory embolization [7, 29].

**Figure 1:**
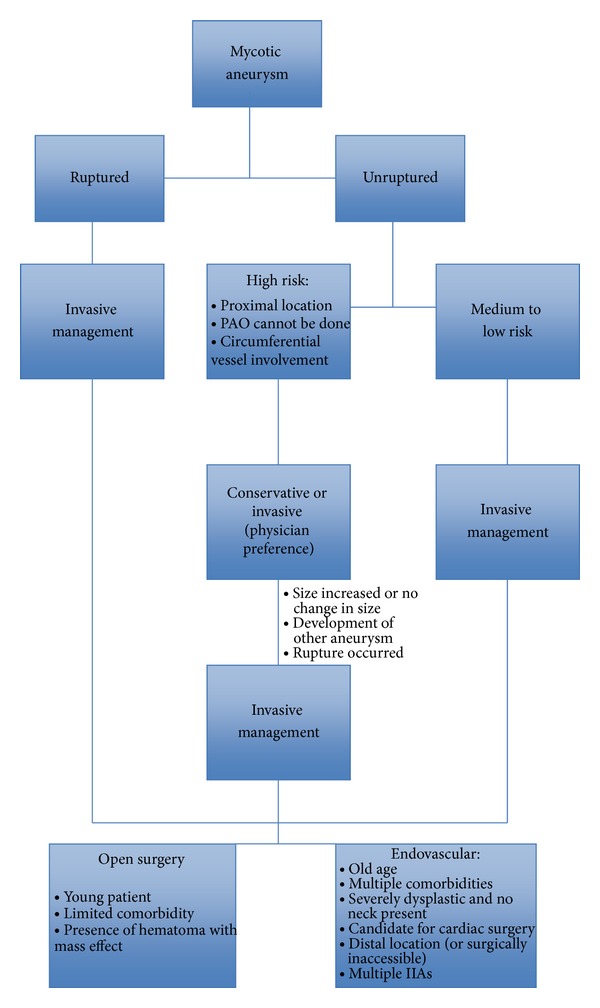
Management algorithm.

Katakura et al. treated pediatric IIAs using NBCA and coils for PAO with no complications from the occlusion of distal MCA branches [46]. Eddleman et al. approached pediatric patients with IIAs that presented with rupture [7]. One patient was treated with PAO using Onyx and another patient was treated by direct coiling followed by Onyx embolization due to persistent filling of the aneurysm on follow-up DSA [7]. The treatment was effective and safe (Tables 3 and 4). For management algorithm, please refer to Figure 1.

**Figure 2:**
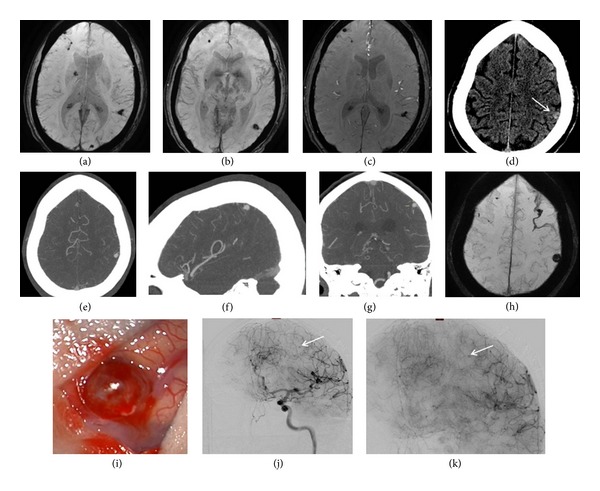
A patient with a history of intravenous drug abuse was admitted to an outside hospital for treatment of endocarditis. MRI at this time demonstrated multiple cerebral septic emboli and mycotic aneurysms (a–c). Two weeks after initiation of antibiotics, the patient had a significant headache and CT scan demonstrated new hemorrhage in the superior parietal lobe (d). The patient was transferred to our hospital for further care, and CTA and MRI at this time demonstrated 2 persistent mycotic aneurysms with hemorrhage surrounding the 7 mm aneurysm arising from the distal cortical branch from the middle cerebral artery (e–h). As the patient required a cardiac valve replacement and would receive full anticoagulation and had a hemorrhage 2 weeks after initiation of antibiotics, the intervention with the ruptured aneurysm was considered the best course of therapy. Due to the distal nature of the aneurysm, microsurgical removal was deemed the best therapy ((i), intraoperative image of cortically based aneurysm). Intraoperative angiogram demonstrated complete resection of the cortically based aneurysm with only the single aneurysm remaining (j, k). Follow CTA demonstrated resolution of the final remaining aneurysm.

At our institution, Thomas Jefferson University Hospital, 4 mycotic aneurysms, 3 of which were associated with arteriovenous malformation and 1 with moyamoya disease, were successfully treated. Complete aneurysm obliteration was achieved in all patients by using Onyx 18 to occlude the aneurysm or to trap the parent vessel, with a procedural related mortality and morbidity rate of 0%. Unfortunately, 2 of our patients died from cardiac complications caused by their endocarditis. The technique that seemed to provide additional safety was the injection just proximal to the aneurysm, thus limiting the distal migration while the filling is taking place. There was neither instances of reflux nor accidental migration of embolic material. There were no recanalization or rebleeding on followup. We conclude that parent vessel trapping with Onyx 18 offers a simple, safe, and effective means of achieving obliteration of distal challenging aneurysms. Avoiding the need for aneurysm catheterization reduces intra-arterial manipulation and thus practically eliminates the risk of aneurysm perforation.  Figure 2 illustrates a case of IIA that was treated by Onyx 18.

## 7. Conclusion

IIAs have a rupture risk of less than 2% [47]. Nevertheless the mortality rate after rupture could reach as high as 80% [21, 48]. In the last decade the flourishing advances in endovascular techniques expanded the scope of its application and have transformed it from a rescue procedure to a first-line treatment as recommended by many authors [28, 42, 48–51]. The majority of the patients with IIAs are quite ill with multiple comorbidities. Therefore, an endovascular approach would be a more suitable treatment option [29]. Unruptured IIAs can be treated with antibiotics and follow-up imaging in 1-2 weeks after therapy. If the aneurysm decreased in size or resolved, then the patient most likely will not need an invasive therapy. Continuation of the antimicrobial in that case would be appropriate while noting that a decrease in size does not correlate with a decrease in the risk of rupture [4]. If the aneurysm is increasing in size or remaining the same, invasive procedures become mandatory. The choice between open surgery and endovascular management depends on a multitude of factors already described above, but the most important are the following: the morphology and location of the aneurysm, whether it is possible or not to sacrifice the parent artery, whether the patient needs or has received valve replacement surgery, and lastly the patient overall health status. Even though there is no head to head RCTs comparing endovascular and open surgery, most infectious aneurysms are being treated by endovascular method [7]. The IIAs of patients considered “strongly immunocompromised” such as those with AIDS, those on chemotherapy, or those on immunosuppressive drugs, have higher rates of growth and rupture [6, 51]. The prognosis of these patients depends on the prompt recognition and early aggressive treatment. Both endovascular and surgical techniques are safe and effective options that have been shown to increase survival when compared to conservative management alone [4].

## Figures and Tables

**Table 1:** Response of aneurysm to medical treatment.

	Disappearance	Decrease in size	No change in size	Increase in size	Additional aneurysm development
Bartakke et al. [20]	29%	18.5%	15%	22%	15%

Corr et al. [24]	33%	17%	33%	17%	

**Table 2:** Characteristics of different agents used in embolization.

Agent	Properties	Advantages	Inconvenience
NBCA	(i) Nonabsorbable, adhesive (ii) Rapid polymerization	(i) High durability (ii) Minimal inflammatory effect	High risk of gluing the microcatheter (instant polymerization)

Detachable coil	(i) New generation soft coil (ii) Hydrogel coated coils (increase in volume once in contact with blood, therefore decreasing initial coil-packing density)	(i) Durable (ii) Decreased risk of rupture (versus old-generation coil)	Risk of rupture (transient increase in pressure while deployment)

Onyx	Nonabsorbable, adhesive	(i) Slow polymerization (ii) Multiple injection from single catheter	(i) Requires familiarity (ii) Requires special catheter

**Table 3:** Aneurysm coiling with or without stent.

GDC*± stent	Modality of treatment	Response
Yen et al. [45]	(i) Helistent 3.5 × 9 mm + GDC for left cavernous carotid (ii) Helistent 4 × 9 mm + GDC for right cavernous carotid	Complete occlusion No complication

Nakahara et al. [1]	(i) 9.2 mm PCA, ultrasoft GDC (ii) 5.7 mm distal left ACA, ultrasoft GDC, treated by PAO	Complete occlusion No complication

Chapot et al. [42] (18 cases)	(i) Nonselective cyanoacrylate (ii) Coil embolization	Complete occlusion No rupture or death

*GDC: Guglielmi detachable coils.

**Table 4:** Results from treatment with Onyx.

Onyx Rx	Location	Treatment/complication
Eddleman et al. [7, 49]: Case 1	M3 4 × 4 mm	Onyx 18, no complication, no filling

Eddleman et al. [7, 49]: Case 2	MCA anterior division 4 × 6 mm	Coiling but persistent filling → Onyx 18 Complications: radiologic distal occlusion due to reflux, but clinically insignificant.

Zhao et al. [43]	(i)11 × 14 mm (ii) P3 of PCA	Onyx 18 under local anesthesia No complications

la Barge et al. [52]	(i) Right parietooccipital artery (fusiform) (ii) Left parietotemporal artery	Onyx 18 No complications Complete occlusion

Our institution	(i) Left MCA at M2 (ii) Left distal ACA (iii) 2 other patients	Complete occlusion 0% combined mortality morbidity
